# Accuracy comparison of tooth volume and mesiodistal diameter measurements for sex dimorphism based on cone-beam computed tomography: a study for the northern Chinese population

**DOI:** 10.1093/fsr/owad004

**Published:** 2023-02-20

**Authors:** Wenqing Bu, Lingling Ji, Mengqi Han, Zixuan Wu, Badr Sultan, Teng Chen, Yu Tang, Yucheng Guo, Fei Wang

**Affiliations:** Key Laboratory of Shaanxi Province for Craniofacial Precision Medicine Research, College of Stomatology, Xi’an Jiaotong University, Xi’an, China; Department of Orthodontics, College of Stomatology, Xi’an Jiaotong University, Xi’an, China; Key Laboratory of Shaanxi Province for Craniofacial Precision Medicine Research, College of Stomatology, Xi’an Jiaotong University, Xi’an, China; Department of Orthodontics, College of Stomatology, Xi’an Jiaotong University, Xi’an, China; Key Laboratory of Shaanxi Province for Craniofacial Precision Medicine Research, College of Stomatology, Xi’an Jiaotong University, Xi’an, China; Department of Orthodontics, College of Stomatology, Xi’an Jiaotong University, Xi’an, China; Key Laboratory of Shaanxi Province for Craniofacial Precision Medicine Research, College of Stomatology, Xi’an Jiaotong University, Xi’an, China; Department of Orthodontics, College of Stomatology, Xi’an Jiaotong University, Xi’an, China; Key Laboratory of Shaanxi Province for Craniofacial Precision Medicine Research, College of Stomatology, Xi’an Jiaotong University, Xi’an, China; Department of Orthodontics, College of Stomatology, Xi’an Jiaotong University, Xi’an, China; College of Medicine and Forensics, Xi’an Jiaotong University Health Science Center, Xi’an, China; Key Laboratory of Shaanxi Province for Craniofacial Precision Medicine Research, College of Stomatology, Xi’an Jiaotong University, Xi’an, China; Key Laboratory of Shaanxi Province for Craniofacial Precision Medicine Research, College of Stomatology, Xi’an Jiaotong University, Xi’an, China; Department of Orthodontics, College of Stomatology, Xi’an Jiaotong University, Xi’an, China; Key Laboratory of Shaanxi Province for Craniofacial Precision Medicine Research, College of Stomatology, Xi’an Jiaotong University, Xi’an, China; Department of Orthodontics, College of Stomatology, Xi’an Jiaotong University, Xi’an, China

**Keywords:** sex estimation, forensic dentistry, teeth measurement, teeth mesiodistal diameter, teeth volume

## Abstract

Sex estimation based on teeth could help to narrow the scope for individual identification. According to the different teeth morphology among both genders, we plan to establish a sex estimation method for the northern Chinese population through mesiodistal diameter and teeth volume measurements and compare the accuracy of the two methods. In this study, measurements were taken from cone-beam computed tomography images collected from 142 males and 140 females aged 21–59 years. The mesiodistal diameter and volume of the left canines and the first molars in both upper and lower jaws were measured and analyzed for suitable coefficients. We selected 80% samples as the training set to set up the logistic regression formulas and 20% as the test set to obtain accuracy. The accuracy of sex estimation by mesiodistal diameter can reach 87.50%, and the volume is up to 78.57%. The measurement of mesiodistal diameter is less time-consuming. This work established and tested a method to estimate sex for the northern Chinese population. Results showed that sex estimation based on the mesiodistal diameter of teeth has higher accuracy than the method based on teeth volume.

## Introduction

Individual identification occupies a critical position in forensic investigation. The aim of the forensic anthropological examination is to find the individual identity, and the sex estimation could be an essential step in the forensic individual identification which can reduce by half the number of suspects or missing persons. The DNA analysis has been widely used in the sex estimation; however, DNA analysis is time-consuming and labour-intensive, which is not advantageous to the timeliness of case. Moreover, many specimens are likely to have been in trace amounts, highly degraded, or mixed which is easy to lead to DNA analysis failure or result error; also, it requires relevant large equipment, which may be not available in remote areas. There are many methods for sex estimation by morphology, including teeth [1], pelvis [2], proximal femora [3], head bones, neck bones [4], and so on. Besides, teeth are the hardest tissues of the human body, resistant to post-mortem degeneration and high temperatures, which made them still preserve a relatively complete shape and structure in harsh environments [5]; on the other hand, genes can determine the size of teeth, which makes teeth have evident sex dimorphism [6]. Therefore, sex estimation by measuring the anatomical structure of teeth has relatively high accuracy and is suitable for forensic practice, especially when complete cranium, pelvis, and DNA information are unavailable. In recent years, sex estimation methods based on dental imaging and dental model measurement have been favoured by researchers and forensic scientists because of their sufficient accuracy.

However, there are few related studies on sex estimation using two-dimensional (2D) images of teeth, mainly based on photos or X-rays [7–10]. Although some studies have shown that sex estimation based on 2D images has a satisfactory accuracy, most related studies showed low accuracy due to factors such as the shooting angle and image resolution [11]. In contrast, tooth sex estimation based on three-dimensional images has received extensive attention recently because of (3D) its accuracy and intuitive data acquisition. Three-dimensional images measurement methods are roughly divided into two types: manual measurement methods [5, 12–17] and digital measurement methods [18–21]. Now, digital technology is widely used in tooth sex estimation because of its high accuracy and relative objectivity. The digital measurement method can not only solve the problem of low accuracy in traditional methods and the inability to retain records, but also can avoid the tedious process of model measurement and obtain huge data information. Hence, it is more suitable for tooth sex estimation. Among them are cone-beam computed tomography (CBCT) images that can provide more information, such as the volume of the root and crown [18, 19, 21].

The mesiodistal diameter has been widely concerned because of its convenience and great performance in sex estimation [13, 22]; on the other hand, some researches focused on the volume indexes which could be obtained from CBCT showed satisfactory result about sex estimation [21, 23], whereas there is few research comparing the effects of the two methods. Some scholars used the teeth linear measurement to conduct sex estimation studies in different populations [15]. However, the results were different, which meant that the accuracy of sex estimation would be affected by ethnicity. In addition, previous studies established population-specific sex estimation formulas to get the high accuracy of sex estimation and verify that ethnicity impacts sex estimation [24, 25]. However, China lacks relevant statistics and research and cannot provide more theoretical support for forensic individual identification.

The aim of the study is to compare the accuracy of the mesiodistal diameter and teeth volume measurements for sex estimation based on CBCT images and establish a proper sex estimation method for the Chinese population.

## Materials and methods

### Sample

The current research was conducted after the approval of the institutional Biomedical Ethics Committee (Xi’an Jiaotong University, No: [2021] 1473). In the present study, a total of 282 individuals aged between 21 and 59 years were selected from the Department of Oral Radiology, Stomatology Hospital of Xi’an Jiaotong University. The age and sex distribution of the included population are shown in Table 1. All the selected individuals were patients who had already performed the CBCTs for initial examination and in equal nutrition and influence of socio-economic status. All CBCTs were taken using the cone-beam X-ray computed tomography system (KaVO 3D eXam I; Charlotte, NC, USA) and exported in DICOM format. The specific inclusion criteria were as follows: (i) there were no teeth with abnormal morphology in the crown and root; (ii) the shape of the crown is complete, and there are no caries for studied teeth; (iii) all the teeth had complete development; (iv) all the permanent teeth (except the third molar) in the maxillary, and mandibular dental arch have erupted, and no deciduous teeth remain; the crowding of teeth in the dental arch could not affect the measurement; (v) no history of severe maxillofacial surgery trauma, deformity, or systemic diseases.

**Table 1:** The age and sex distribution of selected population.

Age (year)	Male (*n*)	Female (*n*)	Total (*n*)
21–30	35	35	70
31–40	36	35	71
41–50	36	35	71
51–60	35	35	70
Total	142	140	282

### Region of interest selection, segmentation, and measurement

**Figure 1:**
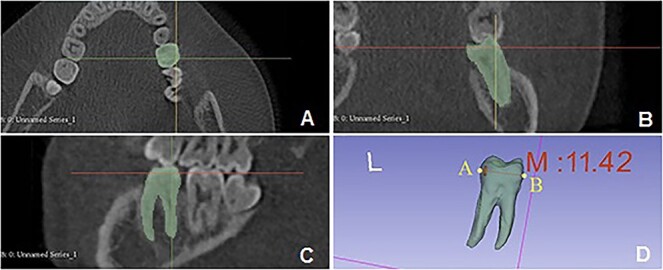
The measurement values in the 3D Slicer software. Make the cone-beamcomputedtomography (CBCT) image segmentation of mandibular first molars as an example: the green part shows the selected area of the teeth in vertical direction; (A) coronal direction (B), and sagittal direction (C). (D) The green model is the segmented mandibular first molars from CBCT image by 3D Slicer, and then to calculate the volume; the distance between the A and B points is the mesiodistal diameter (mean = 11.42 mm).

All CBCT images were screened according to the criteria. CBCT files were imported into 3D Slicer software (version 4.11.20210226; Cleveland, OH, USA) [26] for measurement. The images were manually segmented in the sagittal direction (every 0.4 mm) from the first to the last view of the target tooth. The software could synthesize complete tooth morphology and automatically calculate the volume (Figure 1 and Table 2). We manually marked the most mesial and distal points on the tooth crown in the software according to the CBCT image, then measured the distance between the two points, and got the mesiodistal diameter (the maximum diameter perpendicular to the long axis of the teeth, Figure 1 and Table 2) of the tooth. The teeth were centered on the three planes before taking the measurements. Previous studies have shown that the canines and the first molars have high sexual dimorphism. Furthermore, they can erupt relatively early in human life and hold more tightly in the alveolar bone [5, 6, 15, 27, 28]. So, the maxillary and mandibular canines and first molars were selected as the target teeth to obtain the mesiodistal diameter and the teeth volume.

The results of measurement, the date of CBCT taking, the date of birth, and the sex (male = 1, female = 0) of the subject were recorded.

### Statistical analysis

All the statistical analysis in this experiment was performed using SPSS 18.0 (IBM SPSS Statistics, Armonk, NY, USA). The Kolmogorov–Smirnov test was used to analyze normal distribution. The unpaired *t*-test was used to analyze the influence of sex on the mesiodistal diameter or teeth volume of the dental types. *P* values < 0.05 were considered to indicate statistically significant differences. The Pearson’s correlation test [29] was used to show the relationship between age and mesiodistal diameter, teeth volume, respectively. The data were randomly grouped according to the ratio of the training set:test set = 8:2 (Table 3). The enter logistic regression was used to estimate sex by evaluating each tooth separately and in several teeth combined ways. All the regression formulas need to pass the Hosmer–Lemeshow test. The obtained formula was brought into the test set by SPSS to get the predicted value (male = 1, female = 0). Then the sensitivity, specificity, positive predictive value (PPV), and negative predictive value (NPV) were calculated.

For estimating the inter- and intra-observer variation of measurements, 10% samples (male:female = 1:1) were randomly selected before the experiment and measured by two dentists according to the above method, and another dentist repeated the measurement of the same samples twice at 1-month interval. For determining the level of agreement between repeated measurements by the same examiner and by different examiners, the intraclass correlation coefficient (ICC) was calculated [10]. The ICC is an index that reflects both the degree of correlation and the agreement between the measurements, and ICC >0.9 indicates “excellent” reliability [30]. After the ICC test of the obtained data (*P* < 0.05, ICC test >0.90), the formal experimental measurement was performed.

**Table 2:** Mesiodistal diameter and teeth volume measurements taken in the study.

Measurement	Definition
Mesiodistal diameter measurements	
L23	The mesiodistal diameter of left maxillary canines
L33	The mesiodistal diameter of left mandibular canines
L26	The mesiodistal diameter of left maxillary first molars
L36	The mesiodistal diameter of left mandibular first molars
Teeth volume measurements	
V23	The volume of left maxillary canines
V33	The volume of left mandibular canines
V26	The volume of left maxillary first molars
V36	The volume of left mandibular first molars

**Table 3:** The distribution of the sample studied.

	Training set (*n*)	Testing set (*n*)	Total sample (*n*)
Male	114	28	142
Female	112	28	140
Total	226	56	282

## Result

### Selecting of sex discrimination indicators

#### Reliability analysis

In the reliability analysis, the mesiodistal diameter variation of both inter and intra-observer measurements showed excellent results (ICC = 0.987 and ICC = 0.971, respectively， *P* < 0.05). In the same analysis, the volume variation also performed well (ICC = 0.985 and ICC = 0.970, respectively， *P* < 0.05).

#### Raw data filtering

The descriptive analysis of the measurement both in male and female is shown in Table 4. All sample sets passed the Kolmogorov–Smirnov test (*P* > 0.05) and conformed to a normal distribution. *T*-tests were performed on all the experimental measurements (*P* < 0.05). At the same time, Pearson correlation test was performed for all measured data: all the indicators weakly or not correlated with age (*P* < 0.05).

#### The logistic regression analysis

The logistic regression was performed by the enter conditional method. The volume model showed that the V23 and V33 were significant (*P* < 0.05) for sex discrepancy. Moreover, the model of mesiodistal diameter showed that the L23 and L36 were significant (*P* < 0.05). The analysis above had passed the Hosmer–Lemeshow test (*P* > 0.05).

#### Receiver operating characteristic curve (ROC) curve

**Figure 2:**
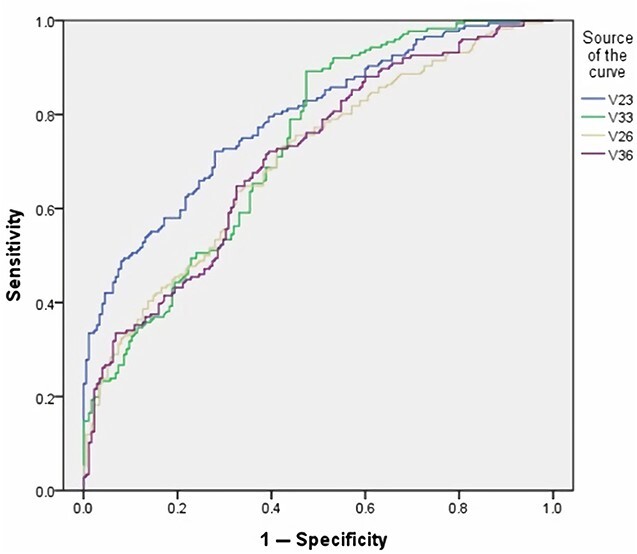
The result of the volume indicators receiver operating characteristic (ROC) curve.

**Figure 3:**
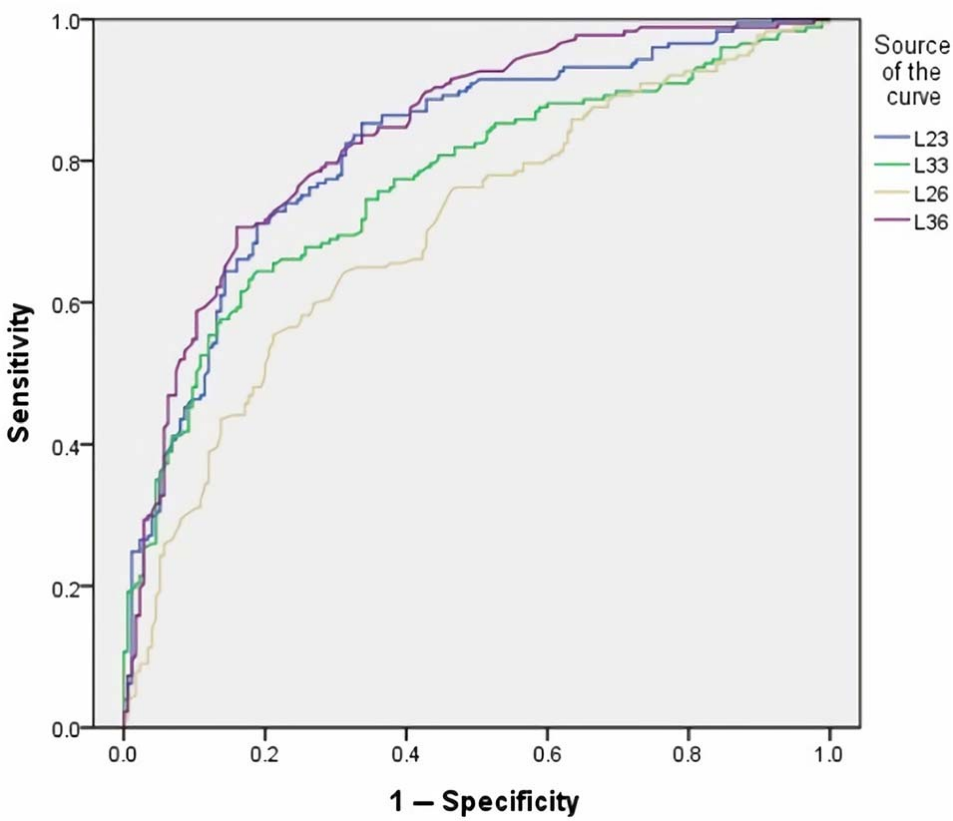
The result of the mesiodistal diameter indicators receiver operating characteristic (ROC) curve.

The ROC curve of volume indicators shows that the V23 and V33 were more effective than V26 and V36 for sex distinction (Table 5 and Figure 2), and the L23 and L36 were more effective than L33 and L26 in mesiodistal diameter (Table 5 and Figure 3) or other volume indicators.

### Obtain the logistic regression formula and test its accuracy, sensitivity, specificity, PPV, and NPV

#### Volume correlation formula

As a result of the above analysis, we selected V23 and V33 as the coefficients of the volume formula. The logistic regression formula and the accuracy of sex estimation are shown in Table 6. Among these, the formula used the left mandibular canines (V33) have the highest accuracy, which is up to 78.57%. Also, the V33 shows the highest sensitivity, specificity, PPV, and NPV among the volume equation.

#### Mesiodistal diameter correlation formula

It can be concluded from the above that L23 and L36 can be used as indicators of the logistic regression formula. The regression formula and the accuracy of sex estimation are shown in Table 6. Furthermore, the formula based on the mesiodistal diameter of left maxillary canines (L23) and left mandibular molars (L36) would achieve the highest accuracy in all the mesiodistal indexes, which is up to 87.50%. Except the sensitivity and PPV of L23 and specificity and NPV of L36, other formula performance test results are better than those of the volume groups.

**Table 4:** The descriptive statistics, Kolmogorov–Smirnov test, *t*-test, and Pearson correlation test results of the research.

	Male				Female				Kolmogorov– Smirnov test		Levene’s test		*T*-test		Pearson correlation test with age	
	Mean	Min	Max	SD	Mean	Min	Max	SD	Kolmogorov–Smirnov Z	*P*-value	*F*	Sig.	*t*	*P*-value	Pearson correlation coefficient	*P*-value
V23 (cm^3^)	0.66	0.42	0.94	0.10	0.54	0.33	0.72	0.09	0.05	0.09	0.01	0.94	(10.81)	0.00^*^	(0.20)	0.001^*^
V33 (cm^3^)	0.59	0.41	1.05	0.10	0.48	0.38	0.68	0.10	0.04	0.20	1.84	0.18	(9.57)	0.00^*^	(0.32)	0.000^*^
V26 (cm^3^)	1.09	0.78	1.59	0.15	0.97	0.54	1.29	0.14	0.05	0.07	0.36	0.55	(6.99)	0.00^*^	(0.20)	0.001^*^
V36 (cm^3^)	1.06	0.77	1.50	0.14	0.93	0.62	1.36	0.14	0.04	0.20	0.02	0.89	(7.53)	0.00^*^	(0.24)	0.000^*^
L23 (mm)	7.89	6.40	9.46	0.64	7.18	6.23	8.89	0.55	0.05	0.08	3.06	0.08	(9.89)	0.00^*^	0.01	0.924
L33 (mm)	7.12	5.24	8.81	0.64	6.60	5.05	7.87	0.51	0.04	0.20	3.51	0.06	(7.56)	0.00^*^	(0.20)	0.001^*^
L26 (mm)	10.46	8.77	12.10	0.72	9.99	8.37	12.21	0.72	0.03	0.20	0.12	0.74	(5.49)	0.00^*^	(0.05)	0.39
L36 (mm)	11.44	9.21	12.88	0.66	10.53	9.02	12.56	0.68	0.05	0.07	0.16	0.69	(11.38)	0.00^*^	(0.04)	0.50

^*^Statistically significant difference (*P* < 0.05). SD: standard deviation.

**Table 5:** The AUC result of each sample group.

Variables^*^	Area	SE	Asymptotic 95% confidence interval	
			Lower bound	Upper bound
V23	0.818	0.024	0.770	0.866
V33	0.783	0.027	0.730	0.837
V26	0.715	0.030	0.655	0.774
V36	0.740	0.029	0.683	0.797
L23	0.799	0.027	0.747	0.851
L33	0.744	0.030	0.686	0.802
L26	0.682	0.032	0.620	0.745
L36	0.834	0.024	0.787	0.881

* The meanings of all variables are given in Table 2. SE: standard error.

## Discussion

Many studies have demonstrated the sexual dimorphism of teeth. It has been reported that the Y chromosome enhances the thickness and size of male teeth by enhancing the formation of amelogenesis and dentinogenesis [31]. Therefore, it is reasonable to estimate sex based on tooth morphology. In addition, teeth, as the hardest tissue of the human body [32], can often be preserved in extreme environments, and in many criminal cases, when the corpse is incomplete [33], dental morphological data can also be used to assist the process of individual identification.

For the studies on sex estimation based on dental morphology, the measurement indicators for tooth normally include mesiodistal tooth diameter, buccal-lingual diameter, diagonal diameter [5], root length [11], and tooth root volume [18]. Among them, the mesiodistal diameter has been widely used for its satisfying accuracy, convenience, and ease of manipulation. Acharya and Mainali [22] have compared the accuracy of sex estimation between the mesiodistal and buccal-lingual diameter and found that the mesiodistal diameter was better for sex estimation. The accuracy of sex estimation (>77%) is higher than that of buccal-lingual diameter (≤64.2%). Kazzazi and Kranioti [13] showed 76.9%–87.9% accuracy in similar studies. Peckmann et al. [12] studied that the accuracy of sex estimation in the mesiodistal diameter of crowns could reach 63.9%–77.6%. As for the tooth position, canines have a longer residence time in the jaw, a higher survival rate, and better sex dimorphism [6, 28]; compared with other tooth types, canines are considered to be the best tooth for sex estimation and are often used as a sample for sex estimation alone [28]. Previous studies have also confirmed the mesiodistal diameter of teeth in specific tooth types, especially the canines [6, 13–16, 27, 28, 34] and the first molar [5, 15, 27] with high sexual dimorphism. The studies of Kazzazi and Kranioti [13], Silva et al. [28], Acharya and Mainali [34], Viciano et al. [15, 16] proved that canines have good sex dimorphism, and the accuracy of sex estimation based on canines can reach 70.2%–81.1%. The first molar is the first permanent tooth erupted in human growth and development. Its roots are long and can exist in the jaw bone early, long-term, and firmly. Zorba et al. [5], Santoro et al. [27], and Viciano et al. [15] pointed out that the accuracy of sex estimation through the first molar is about 75.0%–95.9%, which can be used as a target tooth for sex estimation. Therefore, in the present study, canines and the first molar were selected as the target teeth for sex estimation, and the accuracy of a single tooth can reach 82.14%. These research results were consistent with other similar studies [13, 15–17, 35]. In addition, Thapar et al. [36] measured and compared the mesiodistal diameter of the teeth with the same name on both sides in the study and found that there was no significant statistical difference between the two, suggesting that in sex estimation, when one tooth is missing, the contralateral tooth with the same position can be used instead. Thus, in the present study, though the left tooth was randomly selected as the target tooth for measurement, the estimation method was also applicable for both sides of the teeth. Also, our previous study found that all permanent teeth except for third molars had finished development by the time the person reached 20 years of age [37]. We have selected the sample over 20 years old to ensure the teeth roots were fully developed while checking the roots images.

**Table 6:** Sex estimation equations based on different values.

Independent variable	Equation	SD	Accuracy (%)	Sensitivity (%)	Specificity (%)	PPV (%)	NPV (%)
Volume							
V23	Logit(p) = 14.118 × V23–8.497	0.44	75.00	78.57	71.43	78.57	71.43
V33	Logit(p) = 11.367 × V33–6.003	0.44	78.57	82.14	75.00	82.14	75.00
V23 + V33	Logit(p) = 10.457 × V23 + 4.923 × V33–8.914	0.45	73.21	75.00	71.43	75.00	71.43
Mesiodistal diameter							
L23	Logit(p) = 1.658 × L23–12.471	0.56	82.14	75.00	89.29	75.00	89.29
L36	Logit(p) = 1.798 × L36–19.680	0.21	80.35	92.86	64.29	92.86	64.29
L23 + L36	Logit(p) = 1.501 × L23 + 1.657 × L36–29.380	0.24	87.50	92.86	82.14	92.86	82.14

Logit(p), the probability value obtained by the equation; V23, the volume of the maxillary canine; V33, the volume of the mandibular canine; L23, the mesiodistal diameter of the maxillary canine; L36, the mesiodistal diameter of the mandibular molar; sensitivity, proportion of samples tested as male; specificity, proportion of samples tested as female; PPV, proportion of samples judged to be male by the test that are actually male; NPV, proportion of samples judged to be female by the test that are actually female.

In previous studies, Manhaes-Caldas et al. [21] have measured the crown volume of anterior teeth by CBCT to analyze sexual dimorphism, and the accuracy was 64.1%–83.7%. According to our study, the ROC curve had shown that the maxillary canines volume and the mandibular canines volume had significance in sex estimation (Figure 2); besides, the highest accuracy for sex estimation was to use the mandibular canines (up to 78.57%), followed by the volume of left maxillary canines (75.00%), which was basically similar to the results of Manhaes-Caldas study [21]. However, in the study of Kazzazi and Kranioti et al. [18], the accuracy of sex estimation based on the data of root volume (86.7%–100% for males and 63.5%–100% for females) was slightly higher than the results of our study, which may be due to the whole tooth volume measurement used in this study. The influence of tooth abrasion on the results cannot be ruled out. At the same time, although Van’t Spijker et al. [38] have pointed out that severe tooth wear only accounted for 70% of the 70-year-old population, and the average tooth wear was only 12 μm per year; the population measured between 21 and 59 years old in this study still had an impact on the prediction of the whole tooth volume may due to the physical abrasion.

Currently, it is still unclear which way the volume or mesiodistal diameter measurement has higher accuracy in estimating unknown sex. In our experiment, it was found that the mesiodistal diameter of the single tooth is much better than the volume in sex estimation, and the ROC curve showed that the mesiodistal diameter is more efficient, especially in the maxillary canines and the mandibular molars (Figures 2 and 3). Compared with all combination data, the accuracy of mesiodistal diameter combination data (L23 and L36) for sex estimation was the highest, which can reach up to 87.50%. The other formula performance values (sensitivity, specificity, PPV, and NPV) showed that the L23 was less effective in male, and the L36 was less effective in female; however, it still showed generally better performance in mesiodistal diameter groups, especially using both L23 and L36 (Table 6). According to our result, the mesiodistal diameter of the target tooth was more important than the volume for sex estimation. At the same time, when the 3D Slicer is used to measure the volume of a tooth, it takes about 25 min on average for a professional scholar and about 1 min average to measure the mesiodistal diameter of a tooth, so the volume measurement is more time-consuming and labourious. Therefore, this experiment concludes that the effect of mesiodistal diameter is better than that of volume in sex estimation, and it is more convenient and efficient.

However, crowded teeth, malocclusion, and contact point abrasion may make it challenging to obtain the mesiodistal diameter, and the chance of error may increase; therefore, many researchers have adopted other indexes, such as the diagonal size, neck diameter [5, 11], root length, root volume [23], to reduce the error of sex estimation caused by physiological wear. At the same time, the hip bone, mandible, maxillofacial sinus cavity, and other parts can also be used for sex estimation. Gomes et al. [39] achieved 84% accuracy when using maxillary sinus as the measurement target for sex estimation. Wanzeler et al. [40] have used frontal sinus as the measurement target for sex estimation, and the accuracy rate reached 94.48%. Zheng et al. [41] researched sex estimation using mandibular data with an accuracy of 85.7%–89.0%. The above results are better than our study, and accuracy differences among different anatomical structures can be considered in further studies.

There is still some limitation in our research. We used the total tooth volume as the research index. However, as age increases, the probability of tooth wear increases, which will affect the accuracy of the tooth volume as a sex estimation index. Second, the manual method is used in the tooth measurement process, which is time-consuming, resulting in decreased work efficiency. In future studies, the computer-aided method can be considered to make the measurement much easier and more objective.

## Conclusions

Our research established and tested a complementary method to sex estimation in forensic investigations for the northern Chinese population. Sex estimation based on the mesiodistal diameter of teeth has higher accuracy than the method based on teeth volume. The method also proved to be more practical and effective during operation.

## Authors’ contributions

Wenqing Bu: resources, methodology, data curation, validation, writing-original draft preparation. Lingling Ji: resources, investigation, formal analysis, writing-original draft preparation. Mengqi Han: data curation, resources, formal analysis, writing-original draft preparation. Zixuan Wu: validation, writing-original draft preparation. Badr Sultan: validation, writing-original draft preparation. Teng Chen: conceptualization, methodology, review’editing draft. Yu Tang: conceptualization, methodology. Yucheng Guo: methodology, supervision, project administration, funding acquisition, review’editing draft. Fei Wang: conceptualization, methodology, review’editing draft.

## Compliance with ethical standards

The ethical standards of the institutional and/or national research committee and the 1964 Declaration of Helsinki and its later amendments or comparable ethical standards were followed in the whole process involving volunteers. The current research was conducted after the approval of the Biomedical Ethics Committee of Xi’an Jiaotong University Health Science Center (No: [2021] 1473).

## Disclosure statement

The authors declare no competing interests.

## Funding

This work was supported by Young Science and Technology Star Program of Shaanxi Province of China [2020KJXX-025, to Y-C.G.], Key Research and Development Projects of Shaanxi Province [2020GXLH-Y-008, to F.W.], general project from the field of social development, in Department of Science and Technology of Shaanxi Province under Grant [2019SF-081, to F.W.].
